# Volcanic electrification: recent advances and future perspectives

**DOI:** 10.1007/s00445-022-01591-3

**Published:** 2022-07-29

**Authors:** Corrado Cimarelli, Sonja Behnke, Kimberly Genareau, Joshua Méndez Harper, Alexa R. Van Eaton

**Affiliations:** 1 Department of Earth and Environmental Sciences, Ludwig-Maximilians-Universität München, Theresienstrasse 41, 8033 Munich, Germany; 2 Space and Remote Sensing, Los Alamos National Laboratory, Los Alamos, NM USA; 3 Department of Geological Sciences, The University of Alabama, Box 870338, Tuscaloosa, AL 35487 USA; 4 Sensors for Extreme Environments Lab, Department of Earth Sciences, University of Oregon, Eugene, OR 97403 USA; 5 U.S. Geological Survey, Cascades Volcano Observatory, Vancouver, WA 98683 USA

**Keywords:** Volcanic lightning, Volcano monitoring, Volcanic ash, Explosive volcanism, Lightning detection systems

## Abstract

The electrification of volcanic plumes has been described intermittently since at least the time of Pliny the Younger and the 79 AD eruption of Vesuvius. Although sometimes disregarded in the past as secondary effects, recent work suggests that the electrical properties of volcanic plumes reveal intrinsic and otherwise inaccessible parameters of explosive eruptions. An increasing number of volcanic lightning studies across the last decade have shown that electrification is ubiquitous in volcanic plumes. Technological advances in engineering and numerical modelling, paired with close observation of recent eruptions and dedicated laboratory studies (shock-tube and current impulse experiments), show that charge generation and electrical activity are related to the physical, chemical, and dynamic processes underpinning the eruption itself. Refining our understanding of volcanic plume electrification will continue advancing the fundamental understanding of eruptive processes to improve volcano monitoring. Realizing this goal, however, requires an interdisciplinary approach at the intersection of volcanology, atmospheric science, atmospheric electricity, and engineering. Our paper summarizes the rapid and steady progress achieved in recent volcanic lightning research and provides a vision for future developments in this growing field.

## Introduction

**Fig. 1:**
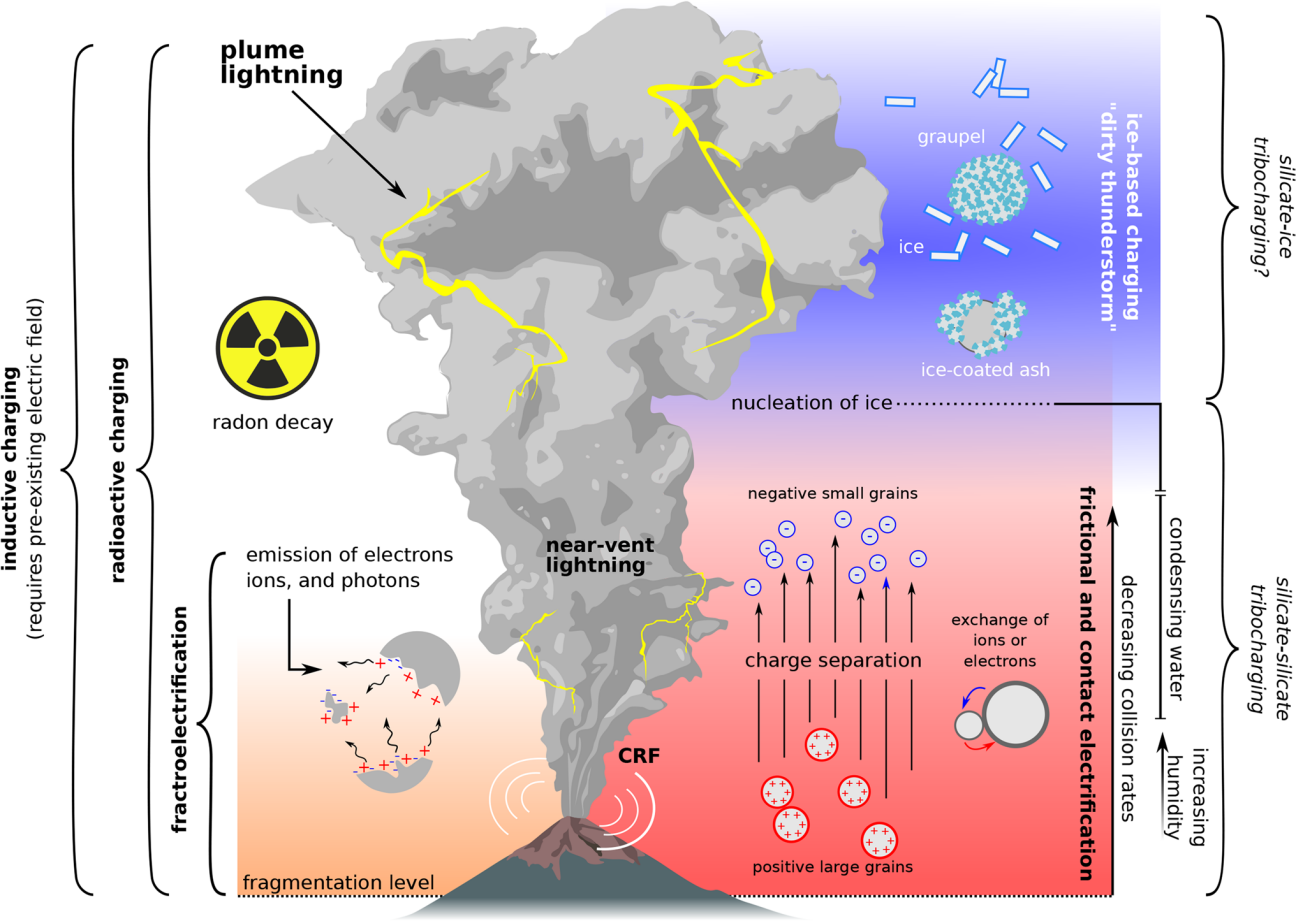
Schematic representation of principal charging mechanisms operating in a volcanic column. Starting at the fragmentation level, material initially charges through fractoelectric processes (orange-lower left). This electrification through fracture continues within the conduit and the gas-thrust region as long as energetic collisions are present. Non-disruptive pyroclast interactions also charge the material through contact and frictional electrification, collectively termed triboelectrification (red-lower right). Importantly, triboelectrification represents a mechanism that can both charge particles and separate charged surfaces of opposite polarity through size-dependent bipolar charging (SDBC). SDBC may be critical in the formation of electric fields required for breakdown. Both fracto- and triboelectrification likely underpin continual radio frequency (CRF) sources and other near-vent electrical activity. As the column develops into a mature plume at altitude, the presence of ice likely catalyzes electrification analogous to that operating within meteorological thunderclouds (blue-upper right). Ice-based charging may generate the large electrical storms observed during large eruptions. Additional mechanisms may operate within columns including the radioactive decay of elements like radon and inductive charging driven by pre-existing electric fields. Note: image is not to scale and only shows location of electrification mechanisms relative to each other

Volcanic ash plumes can produce intense electrical activity and lightning storms (Fig. 1; Rakov and Uman 2003; Mather and Harrison 2006; James et al. 2008; McNutt and Thomas 2015; Cimarelli and Genareau 2022). Increasing reports of volcanic lightning and instrumental detection following the eruptions of Augustine 2006, Redoubt 2009 and Eyjafjallajökull 2010 (Bennet et al. 2010; Thomas et al. 2010; Behnke et al. 2013) show that electrification is ubiquitous in ash plumes. The rapidly evolving knowledge of electrification phenomena at volcanoes has been proceeding hand in hand with technological advances in field-based detection and laboratory experiments as well as growing interest from the public. Social media allows global sharing of photos and videos documenting visible electrical activity in volcanic plumes even in the absence of dedicated instruments. This development has increased awareness that volcanic lightning is not just a striking feature associated with explosive eruptions but provides a new avenue to quantify the dynamics of explosive eruptions and their volcanic plumes.

Lightning detection is emerging as a new tool in the portfolio of eruption monitoring techniques. Volcanic lightning can be a near-real time indicator of explosive ash emissions due to rapid detection with remote instruments (including satellite-based sensors) and in conditions of poor visibility. Furthermore, advances in the satellite- and ground-based instruments routinely used to track lightning storms (e.g., Cummins and Murphy 2009; Rakov 2013; Nag et al. 2015) can also be applied to eruption monitoring (Poland et al. 2020). As a result, in the last few decades, multi-disciplinary research efforts have gained momentum in refining our understanding of this phenomenon and its link to eruption dynamics and hazards. The intrinsic difficulty of probing the interior of a volcanic plume emphasizes the importance of linking field and remote sensing observations to constrained laboratory experiments, where the basic mechanisms of gas-particle electrification can be systematically mapped. In addition, several key observations made possible by laboratory experiments also hold true for the natural systems, thus meaning that a many-fold approach is beneficial for fully understanding volcanic electrification processes.

This perspective piece briefly summarizes the state of the art in plume electrification and volcanic lightning gained in the last decade and projects future research on this topic outlining the expected goals in the field of volcano monitoring and the wider context of explosive volcanic eruptions.

## Detection and characteristics of volcanic lightning

In the past decade, volcanic lightning research has benefited from the proliferation of a variety of lightning detection methods (Behnke and McNutt 2014). 2-D lightning locations from regional or long-range radio frequency (RF) sensor networks have enabled studies of volcanic lightning and plume charging mechanisms across the globe (e.g., Arason et al. 2011; Hargie et al. 2018; Van Eaton et al. 2016; Prata et al. 2020). 3-D very high frequency (VHF) Lightning Mapping Arrays (LMA) have been used for detailed study of volcanic lightning and the charge structure of volcanic plumes (Behnke et al. 2013; 2014). Photographs (Behnke et al. 2012; Van Eaton et al. 2022) and high-speed videos (Cimarelli et al. 2016) have provided a complementary ground truth to the RF measurements, thus providing the necessary context to define volcanic lightning types. From space, the Geostationary Lightning Mapper onboard GOES-16 offers persistent monitoring of volcanoes in the western hemisphere (Schultz et al. 2020). In addition, novel lightning monitoring instrumentation (Aizawa et al. 2011; 2016; Vossen et al. 2021; 2022) and sometimes serendipitous uses of established volcano monitoring instruments (Haney et al. 2018; 2020), further contribute to our volcanic lightning knowledge.

**Fig. 2:**
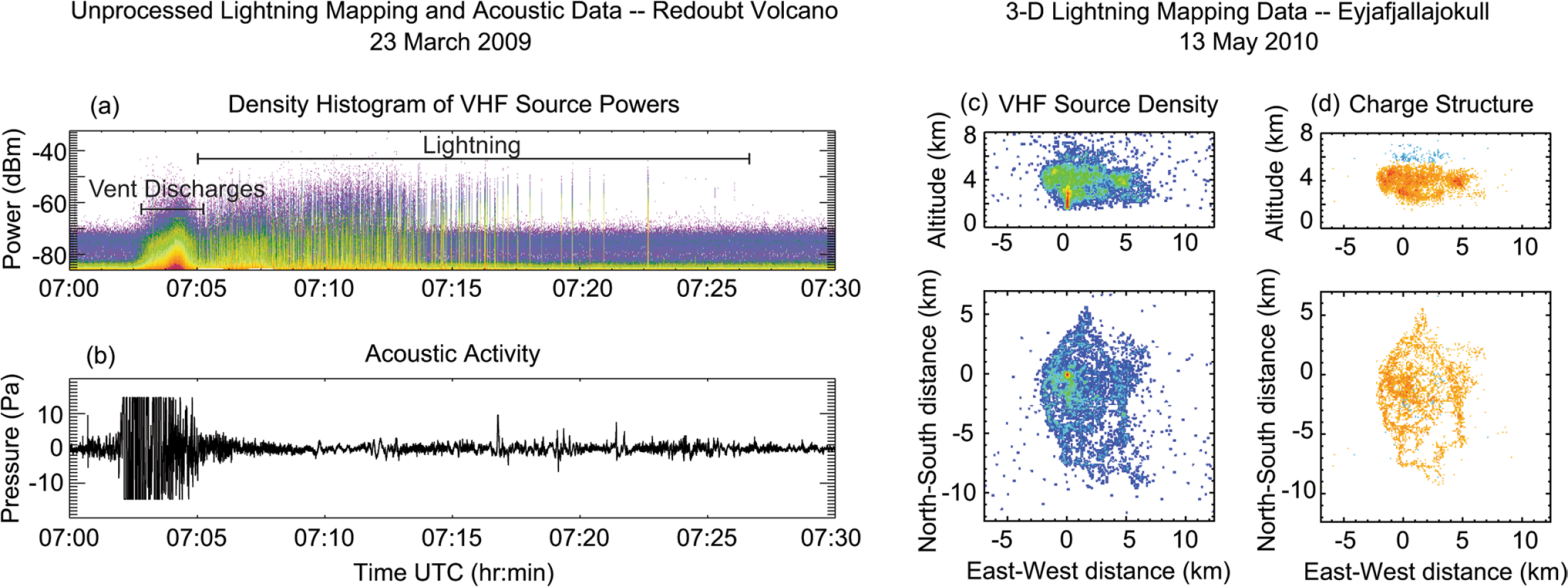
**a**–**b** Unprocessed LMA data and acoustic activity from an explosive event on 23 March during the 2009 eruption of Redoubt Volcano, adapted from Behnke et al. (2013). **a** Density of VHF source powers. Electrical activity begins as a burst of high-rate vent discharges at the onset of the explosive event. This is followed by lightning, which becomes increasingly intermittent over time. **b** Acoustic activity; data were shifted in time to account for travel time delay. **c**–**d** 3-D LMA data during the second explosive period of the 2010 eruption of Eyjafjallajokull, adapted from Behnke et al. (2014). **c** Density of located VHF sources. **d** VHF sources colored according to the inferred charge polarity (blue is negative, orange is positive) based on LMA data analysis. The top plots in **c**–**d** show altitude vs East–West distance, while the bottom plots show the plan position of the source locations

With increasing lightning observations, we have learned how the electrical activity evolves over the course of an explosive eruption, gaining insights into the charge structures of volcanic plumes. Generally, volcanic electrical activity manifests as vent discharges and lightning. Vent discharges typically occur in a high-rate burst within the gas-thrust region of an ash-rich plume (Thomas et al. 2010; Behnke et al. 2018), producing a characteristic VHF signal referred to as continual radio frequency (CRF; Fig. 2) (Thomas et al. 2007; Behnke et al. 2012, 2013). Vent discharges are hypothesized to be a form of streamer discharge (Behnke et al. 2018, 2021), rather than full-fledged lightning. Recent observations have shown that vent discharges commence before large-scale charge separation develops in the eruption column (Behnke et al. 2018) and that they may be enabled by the reduced atmospheric electric breakdown threshold in the near-vent region resulting from the propagation of shock waves (Méndez Harper et al. 2018; von der Linden et al. 2021). Vent discharges are correlated with the intensity of explosions (Smith et al. 2020, 2021) in which magma fragmentation and particle comminution occur (Smith et al. 2018a).

Lightning can occur throughout an eruption column, in a continuum of flashes ranging in length from tens or hundreds of meters proximal to the vent (Behnke et al. 2018; Aizawa et al. 2016; Thomas et al. 2010), to tens of kilometers in a fully developed ash cloud (Behnke et al. 2013, 2014). The locations and characteristics of lightning reveal information about the charge structure of a plume and can aid in inferring eruption styles (Fig. 1). In a typical explosive eruption, lightning often occurs within seconds of the onset of an explosion, in the gas-thrust region, where turbulence leads to a heterogeneous (Behnke et al. 2013; Aizawa et al. 2016; Cimarelli et al. 2016) and high-density charge structure (Haley et al. 2021). Over time, and in concert with the convection and eventual lateral spreading of the ash cloud, lightning increases in size, and horizontally stratified charge layers develop (Behnke et al. 2013, 2014; Behnke and Bruning 2015). Lightning rates have been correlated with increases in mass eruption rate, showing that particle acceleration, turbulence, and plume height/extent may all contribute to charging (Cimarelli et al. 2016; Hargie et al. 2018; Smith et al. 2021). In addition, lightning has been observed in association with pyroclastic flows, providing a means to differentiate changes in eruptive behavior (Van Eaton et al. 2016; Schultz et al. 2020).

Charging in volcanic plumes may also be actively detected using satellite GNSS networks such as GPS (Méndez Harper et al. 2019). Dense plumes of volcanic ash can attenuate GPS signals, providing information on microphysical and charge properties of the plumes (Aranzulla et al. 2013; Larson 2013; Grapenthin et al. 2018).

## Electrification mechanisms

Lightning and other electrical phenomena associated with eruptions arise from processes that generate, separate, and concentrate charge within volcanic plumes. Electrification mechanisms evolve over the temporal and spatial development of volcanic plumes, and vary greatly with eruption style and intensity. The key mechanisms are broadly characterized by silicate charging and ice charging. Other charging mechanisms have been proposed which include the effects of magma-water interaction and water vaporization (Blanchard 1964; Björnsson et al. 1967; Büttner et al. 1997), the ambient electric field (Pähtz et al., 2010), and the radioactive decay of ash (Aplin et al. 2014) and gas (Nicoll et al. 2019).

### Silicate charging

The common occurrence of electrical activity near to the vent implies that silicate charging processes begin at the earliest onset of eruption, within the vent or even the upper conduit. One process by which pyroclasts may charge in the absence of an ice phase involves the release and capture of ions (or electrons) during fragmentation and subsequent disruptive particle–particle collisions (Dickinson et al. 1981). Laboratory experiments suggest that released charge carriers may then be redistributed among particles of different aerodynamic diameters to produce large-scale charge separation required for breakdown (James et al. 2000).

Within the conduit and the near-vent region, non-disruptive collisions may also produce charging through triboelectrification, which encompasses contact and frictional electrification (Aplin et al. 2014; Méndez Harper and Dufek 2016). Triboelectricity occurs when two surfaces collide or rub and then separate. Despite being the oldest-known manifestation of electricity, there is no consensus on the charge species exchanged upon contact. Some authors suggest that tribocharging is driven by the exchange of electrons trapped in unfavorable energy states (Forward et al. 2009a,b,c), whereas others suggest that electrification may reflect the transport of ions (possibly water ions) across surfaces (Gu et al. 2013; Xie et al. 2016). Additional experiments (Baytekin et al. 2011) have revealed that microscopic bits of material are exchanged between surfaces during contacts. Such findings imply that triboelectricity and fractoelectricity may be related.

Triboelectric charging of granular materials often results in smaller, negatively charged particles and larger, positively charged particles (Forward et al. 2009c; Waitukaitis et al. 2014; Lee et al. 2015; Méndez Harper et al. 2021b). This size-dependent bipolar charging may be critical for generating discharges in proximal volcanic jets and other dusty flows (Forward et al. 2009b; Méndez Harper et al. 2018, 2021a) as particles of different sizes and opposite charge become separated through turbulence (Cimarelli et al. 2014; Behnke and Bruning 2015) or particle sedimentation (Miura et al. 2002; Zheng et al. 2003; Harrison et al. 2016).

The relationship between silicate charging and volatiles is poorly understood. Although small amounts of water may be responsible for frictional electrification through ion transfer, there is evidence that larger amounts of liquid water may effectively shut off tribocharging in a granular flow. For instance, experiments by Méndez Harper et al. (2020) showed that the magnitude of charge on agitated volcanic ash decreases with increasing humidity, consistent with the experimental findings of Toth et al. (2017). Observationally, at least one of the Alaskan eruptions of Bogoslof volcano in 2017 (event 29) produced less lightning after seawater inundated the vent (Van Eaton et al. 2020).

Overall, the current experimental and observational data suggests that fracto- and triboelectric charging are likely electrification mechanisms within the conduit and in near-vent region. These two mechanisms may be responsible for the production of electrical activity occurring near to the vent. However, additional efforts are required to determine (1) the relative efficiency between both mechanisms and whether they operate synergistically; (2) the manner in which fractocharging redistributes charge among fragments; and (3) whether or not fracto- and triboelectric charging are fundamentally end members of a broader fractotriboelectrification mechanism.

### Ice charging

Although large amounts of water at the eruptive vent may be detrimental to charging, electrification may be reinvigorated by the presence of ice as volcanic plumes rise into freezing levels of the local atmosphere (generally colder than –20 °C; Genareau et al. 2018; Maters et al. 2019, 2020). Numerical simulations show that phreatomagmatic eruptions, which incorporate external water (e.g. from lakes, glaciers, hydrothermal systems), develop a mixed-phase region within the turbulent core of the upper plume. This region contains liquid water, cloud ice, and graupel/hail (Williams and McNutt 2005; Van Eaton et al. 2012, 2015, 2022), triggering thunderstorm-like ice charging mechanisms (Saunders 2008). These conditions may explain the impressive rates of volcanic lightning rivaling supercell thunderstorms (e.g., Arason et al. 2011; Behnke et al. 2013; Prata et al. 2020; Van Eaton et al. 2020). “Dry” magmatic eruptions can also produce ice in upper levels of the plume (Van Eaton et al. 2016), but lack sufficient liquid water to sustain a vigorous mixed-phase region. Mounting evidence suggests that the presence of both ice and sustained updrafts in tall plumes contribute to abundant, higher-current lightning discharges detectable by long-range sensors (Arason et al. 2011; Woodhouse and Behnke 2014; Van Eaton et al. 2020, 2022).

## Evidence and effects of lightning in ashfall deposits

Lightning interacts with and modifies volcanic particles, providing a possible means of physically documenting the occurrence of lightning through the ashfall deposits. Several types of modified ash have been observed. Lightning-induced volcanic spherules (LIVS) range from 1 to 100 μm in diameter and can be solid or hollow (Genareau et al. 2015). Solid LIVS form from a single ash particle while hollow LIVS form from detachment of an expanding gas bubble from a larger pumiceous particle (Genareau et al. 2019a). Their upper size limit is compatible with the timescale of ash melting by lightning discharges (Wadsworth et al. 2017). Lightning-generated pumiceous particles observed so far are differentiated from primary fragmentation textures by their lack of microlites and smooth, glassy outer surfaces (Genareau et al. 2019a). Fulgurites in volcanic deposits are rare, probably due to the difficulty in finding and recognizing them in the field (Clocchiatti 1990; Castro et al. 2020).

## Experimental generation of lightning and lightning simulation experiments

### Shock-tube experiments

Particle-laden jets generated by rapid decompression in a shock-tube under controlled environmental conditions produce electrical discharges by self-charging mechanisms (Cimarelli et al. 2014). Experimental data show that vent overpressure, mass of solid ejected, and proportion of fine particles affect discharge characteristics. While the proportion of fine particles positively correlates with the total number of discharges, initial pressure and ejected mass may control the discharge size (Gaudin and Cimarelli 2019). Furthermore, increasing the total water content of the mixture dramatically reduces the occurrence of discharges in the jet (Stern et al. 2019).

### Current impulse experiments

Laboratory modification of volcanic ash has been achieved through high-current (7–100 kA) electrical impulse experiments, which recreate the temperatures (~ 20,000 ℃) (Genareau et al. 2017) and magnetic fields generated in the plasma channel of natural lightning exceeding by orders of magnitude the strength of the Earth’s magnetic field. These experiments produce textures in pseudo-ash samples like those found in ashfall deposits (Genareau et al. 2020) and show that particles proximal to the lightning channel may be disassociated, melted, and fused together (Genareau et al. 2017). Although the radial zone of the channel suitable for melting is only 10% of the entire channel volume (Genareau et al. 2017), these effects will destroy or fuse together fine ash particles (Genareau et al. 2019a) and potentially modify portions of larger pyroclasts in the plume. In the experiments, melted particles tend to lack microlites and display mingled heterogeneous glass compositions with zones of element enrichment (Woods et al. 2021). Particles may also vesiculate by exsolution of residual volatiles in the glass phase, analogous to natural lightning-induced pumiceous particles (Genareau et al. 2019a). Additionally, the magnetic properties of the particles change, showing increased magnetization (Genareau et al. 2019b), similar to locations struck by cloud-to-ground lightning (Cox 1961; Sakai et al. 1998; Verrier and Rochette 2002).

## Lightning detection in volcano monitoring

**Fig. 3:**
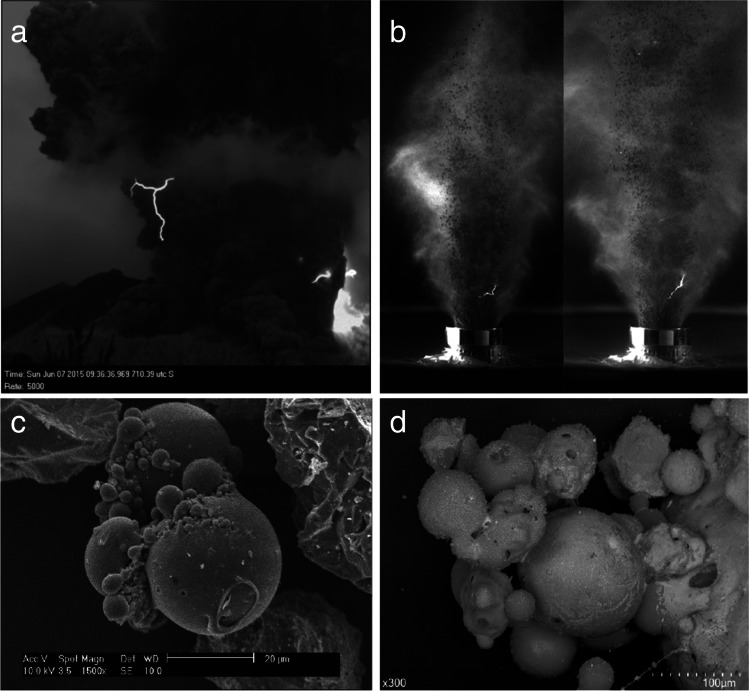
Nature and experiments in comparison. **a** High-speed (5000 fps) video frame of near-vent lightning at Sakurajima volcano (Japan). **b** High-speed (30 k fps) video frames of discharges in shock tube experiments with gas-ash particle mixtures (note centimetric scale on the nozzle). **c** Secondary electron images of spherule aggregates from the 2009 Redoubt eruption (modified from Genareau et al. 2015). **d** Agglutinated LIVS generated from natural phonolitic volcanic ash in electrical (35 kV, 300A) experiments

As with ordinary electrical discharges, volcanic lightning can pose a direct hazard to life and property (McNutt and Thomas 2015; Van Eaton et al. 2022), including destroying volcano monitoring stations. Few countries account for lightning threats in their long-term volcanic hazard maps, with the notable exceptions of Iceland and Chile. For example, hazard maps for Katla volcano indicate a volcanic lightning zone within 40 km of the vent (Icelandic Civil Protection 2017) (Fig. 3).

**Fig. 4:**
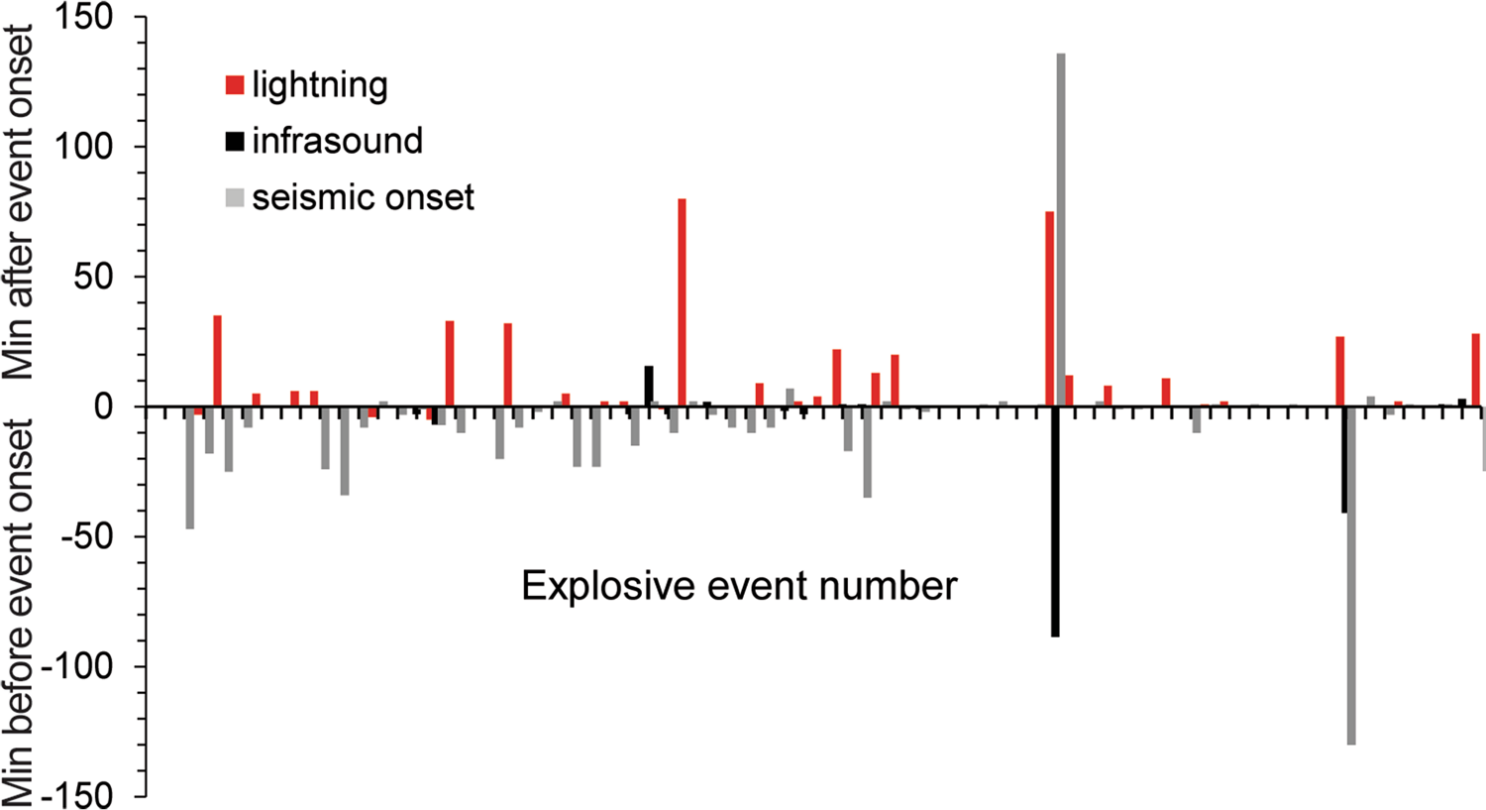
Timing of globally detected lightning compared to remote seismic and infrasound observations during the 70 explosive events from Bogoslof volcano, Alaska, from 2016–2017. Note that lightning tends to lag the estimated onset of explosive activity by minutes to tens of minutes (reflecting timescales of eruption intensification, plume rise, and ice formation), although in some cases, lightning was the earliest sign of eruption. Adapted from Van Eaton et al. (2020)

Volcanic lightning can also help detect and characterize explosive activity to keep aircraft out of harm’s way. Its operational use has evolved since early applications at Mount Spurr, Alaska, in 1992 (Paskievitch et al. 1995) and Grímsvötn, Iceland, in 1998 (Arason et al. 2013 and references therein). In recent years the USGS Alaska Volcano Observatory has started integrating global lightning detection into automated eruption alerts (Coombs et al. 2018). During the 2016–2017 eruption of Bogoslof volcano in the remote Aleutian Islands, lightning provided the earliest indication of ash-rich plumes in some cases (Fig. 4). However, there were also important limitations—lightning was only detected in ash plumes sustained above the local atmospheric freezing level. Thus, fewer than half of the 70 explosive events from Bogoslof created lightning that was measurable by long-range sensors (Van Eaton et al. 2020). An additional limitation is that it can be difficult to distinguish volcanic lightning from background storms in tropical regions with abundant meteorological lightning, such as the Philippines and Indonesia (Hargie et al. 2018). In the future, this challenge may be addressed by tracking spatial changes in lightning locations through time to identify the vent location and eruptive processes (Arason et al. 2013; Smith et al. 2018b; McKee et al. 2021a,b).

Despite these limitations, volcanic lightning detection fills an important gap in the suite of monitoring tools used by volcano observatories. Each of the existing monitoring approaches has strengths and weaknesses, depending on the level of in situ monitoring at a given volcano (Coombs et al. 2018). The value of long-range lightning sensors is that they can assist the early recognition of ash-rich eruptions that send a plume above freezing altitudes (Fig. 4). By comparison, there can be several to tens of minutes between satellite images available in real time and views can be obscured by cloud cover (Poland et al. 2020; Engwell et al. 2021). Infrasound signals may be affected by local wind or complex atmospheric propagation, and seismic data may not clearly distinguish eruptive and pre-eruptive ground motion (Coombs et al. 2018). Globally detected lightning is becoming increasingly integrated at volcano observatories and volcanic ash advisory centers around the world, including in Kamchatka (Shevtsov et al. 2016) and Argentina (Baissac et al. 2021). Recent recommendations for operational volcano monitoring in the USA have also been updated to include volcanic electrification (Flinders et al. under review).

## Future directions in volcanic lightning research

Looking ahead, new laboratory experiments would be valuable to detail charging mechanisms under the variable conditions dictated by volcanic activity and the surrounding environment. Parameters such as grain size distribution, grain composition, temperature, pressure, and volatile content (H_2_O, SO_2_, and other species) should be further explored. Although previous experiments have provided an initial framework, the influence of volcanic particles on charging and discharge characteristics should be tested at the laboratory scale to improve interpretations of the remotely sensed datasets. We expect these experimental results may be combined with existing lightning models from meteorology to build electrification modules into high-resolution numerical simulations of volcanic plumes and their electrical behavior. Moreover, the impacts of lightning on volcanic plumes and the phases within them leave many avenues for further exploration. It has been shown that lightning alters volcanic ash properties, but it is not yet known how these physical, chemical, and magnetic changes may influence volcanic ash on a broader scale. There is also a potentially measurable effect on the atmospheric properties surrounding the discharge channel. The rapid melting of solid phases coupled with reduction/oxidation of volatile components in the volcanic plume may produce a number of unique chemical species important for various Earth system processes, including biologic activity (Navarro-González and Segura 2001).

Volcanic lightning research is rapidly moving forward through multi-disciplinary studies and technological advances. Research efforts in the coming decade will continue to extend volcanic lightning observations to a wider range of explosive activity following a multi-instrumental approach. Combining the various ground-based and satellite-based remote sensing instruments will be key to constraining lightning properties, plume dynamics, and microphysics. Paired with long-distance lightning detection, the development of miniaturized and cost-effective near-field sensors to simultaneously monitor lightning properties (e.g., electromagnetic fields, infrasonic signals) and eruption plume properties (e.g., SO_2_ content, grain size distribution) will enable more direct analysis of smaller eruptions where lightning may go undetected by global networks. Each of these developments will support our ability to incorporate near-real time analysis of lightning data to characterize volcanic plumes in operational settings. Constraining eruption source parameters such as plume height, mass eruption rate, and ash/water content using volcanic lightning will ultimately play a growing role in forecasting volcanic ash hazards in the atmosphere and on the ground.

The study of volcanic lightning has, so far, bridged the disciplines of geology, atmospheric science, atmospheric electricity, remote sensing, and electrical engineering. In years to come, it will likely be relevant to many other scientific fields due to the complexity of this natural phenomenon.
